# PMTED: a plant microRNA target expression database

**DOI:** 10.1186/1471-2105-14-174

**Published:** 2013-06-03

**Authors:** Xiuli Sun, Boquan Dong, Lingjie Yin, Rongzhi Zhang, Wei Du, Dongfeng Liu, Nan Shi, Aili Li, Yanchun Liang, Long Mao

**Affiliations:** 1 Key Laboratory of Symbol Computation and Knowledge Engineering of Ministry of Education, College of Computer Science and Technology, Jilin University, Changchun 130012, China; 2 Institute of Crop Science, Chinese Academy of Agricultural Sciences, Beijing 100081, China

**Keywords:** MiRNA, Microarray, Meta-analysis, Stress response

## Abstract

**Background:**

MicroRNAs (miRNAs) are identified in nearly all plants where they play important roles in development and stress responses by target mRNA cleavage or translation repression. MiRNAs exert their functions by sequence complementation with target genes and hence their targets can be predicted using bioinformatics algorithms. In the past two decades, microarray technology has been employed to study genes involved in important biological processes such as biotic response, abiotic response, and specific tissues and developmental stages, many of which are miRNA targets. Despite their value in assisting research work for plant biologists, miRNA target genes are difficult to access without pre-processing and assistance of necessary analytical and visualization tools because they are embedded in a large body of microarray data that are scattered around in public databases.

**Description:**

Plant MiRNA Target Expression Database (PMTED) is designed to retrieve and analyze expression profiles of miRNA targets represented in the plethora of existing microarray data that are manually curated. It provides a Basic Information query function for miRNAs and their target sequences, gene ontology, and differential expression profiles. It also provides searching and browsing functions for a global Meta-network among species, bioprocesses, conditions, and miRNAs, meta-terms curated from well annotated microarray experiments. Networks are displayed through a Cytoscape Web-based graphical interface. In addition to conserved miRNAs, PMTED provides a target prediction portal for user-defined novel miRNAs and corresponding target expression profile retrieval. Hypotheses that are suggested by miRNA-target networks should provide starting points for further experimental validation.

**Conclusions:**

PMTED exploits value-added microarray data to study the contextual significance of miRNA target genes and should assist functional investigation for both miRNAs and their targets. PMTED will be updated over time and is freely available for non-commercial use at http://pmted.agrinome.org.

## Background

MiRNAs are emerging key regulators in plant development and stress responses [1] and have been identified in a number of plants as demonstrated in miRBase [2]. Plant genomes contain ~200 miRNAs, each with an average of 2–3 target genes, many of which are important transcription factors. Study of miRNA target gene expression patterns may provide novel clues about the functions of miRNA-target interactions, which may be already available in the large amount of existing microarray experiments. Microarray technology has been a major tool for genome wide transcriptome analysis and is extensively used in the past two decades. Thousands of experiments were performed and a humongous amount of data has been generated and deposited in databases such as ArrayExpress [3,4] and Gene Expression Omnibus (GEO; [5]). These experiments study important biological processes such as biotic stress, abiotic stress, various tissues and developmental stages and involve many plant species, including important crops such as rice, maize, wheat and soybean.

The large spectrum of biological conditions and species covered by microarray experiments render them valuable information to be explored by bioinformatics efforts. For example, Pathogen, as a relational database for annotation of all identified signal transduction mechanisms during plant-pathogen interactions, has incorporated gene expression data from *Arabidopsis thaliana* microarray experiments enabling easy access to specific genes regulated upon pathogen infection or elicitor treatment [6]. Among the genes on the microarrays are targets of miRNAs. However, without further processing, these data are difficult to reach by biologists. Toward this end, we present PMTED, a manually curated plant miRNA target expression database that comprises query, analytical, and visualization functionalities for more than 5,000 miRNA targets under several hundred experiments for 12 plant species. In addition, meta-data from 92 experiments were curated and can be searched and browsed so that novel hypotheses of miRNA and target interactions can be generated for further validation. PMTED is designed (1) to facilitate the plant miRNA research by making the large amount of target expression information available; and (2) to provide a portal to facilitate the transfer of model plant resource such as those of Arabidopsis and rice to less studied crop species such as wheat and maize.

## Construction and content

### Datasets and processing

#### Microarray data

**Figure 1:**
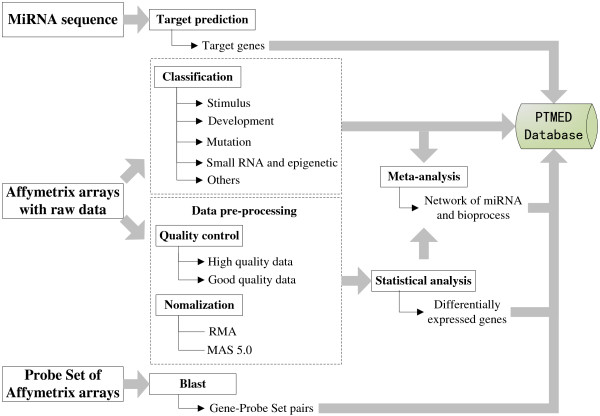
PMTED data processing workflow.

Microarray experiments performed on Affymetrix platform were retrieved from Gene Expression Ominibus (GEO). Experiments without raw intensity data in the CEL format or no biological duplications were excluded. Eventually we picked a total of 3,492 arrays from 311 experiments. For easy query and browse purposes, we classified these experiments into 5 classes: stimulus, development, mutation, small RNA and epigenetic and others, according to their experimental subject (Additional file 1: Table S1). The raw data were then pre-processed using Robin [7], an R/BioConductor-based program (Figure 1). Data normalization was performed using the robust multi-array average (RMA, [8]) and Affymetrix Microarray Analysis Suite 5.0 (MAS 5.0) which calculated absent/present call and attached *p*-values for each probe set. Quality control was performed using MA plots, with a threshold of no more than 10% of the genes showing a greater than two-fold change for high quality datasets. The remaining data was considered as “good quality”. Probe Sets annotation information and target sequences were obtained as Affymetrix supporting documents (http://www.affymetrix.com/estore/) and were linked to either locus identifiers for species with sequenced genomes such as Arabidopsis and rice, or other identifiers such as PlantGDB names of wheat EST contigs (http://www.plantgdb.org/).

#### MiRNA target prediction

A total of 2,393 miRNA mature sequences were downloaded from Plant MicroRNA Database (PMRD, http://bioinformatics.cau.edu.cn/PMRD/) [9]. They were used to predict target genes among gene models or CDSs of species with sequenced genomes and the most updated cDNA sequences or PlantGDB contigs for species without annotated gene sets. An in-house program written in Perl scripts was generated with target quality evaluation criteria as suggested by Allen *et al.*[10], in which mismatched bases were penalized based on their locations in the miRNA/target alignment. In comparison with published miRNA targets, we adopted a relative stringent cut-off threshold score of ≤ 4 and E-value = 10. Under this condition, a total of 5,449 target genes were predicted for 1,897 miRNAs (Table 1). Despite that fewer targets were obtained by our pipeline when compared with the psRNATarget program [11], our dataset contains more degradome validated targets as shown in rice [12] (Additional file 2: Figure S1), indicating that our pipeline generated fewer false positives.

**Table 1:** Statistics of miRNAs, targets, series, and microarrays in PMTED

**Species**	**miRNA related**		**Experiment related**	
	**#miRNA**^**1**^	**#Target**	**#Series**	**#Array**
*Arabidopsis thaliana*	204	548	167	1959
*Citrus sinensis*	50	244	3	30
*Glycine max*	132	402	23	245
*Gossypium hirsutum*	45	133	2	20
*Medicago truncatula*	355	1121	11	105
*Oryza sativa*	411	880	43	466
*Pupolus trichocarpa*	206	369	2	10
*Saccharum officinarum*	31	80	2	20
*Solanum lycopersicum*	31	85	9	1140
*Tricticum aestivum*	74	1084	14	160
*Vitis vinifera*	121	148	4	36
*Zea mays*	237	355	31	327
Total	1897	5449	311	3492

^1^These miRNAs confer predicted targets.

#### Data curation and classification

We further screened the annotation information of high quality experiments. A total of 92 experiments from nine species of 202 sample comparisons were selected and classified into three major bioprocesses: abiotic stress, biotic stress, and development (Additional file 1: Table S1). These experiments were further divided into subgroups according to their commonality in experimental conditions that were represented by unique terms. A total of 55 terms were extracted from the annotation, including 25 abiotic stress terms (such as drought, salt, and acid), 20 biotic stress terms (such as rice stripe virus, powdery mildew and fungus), and 10 development terms (such as leaf, root and stem) (Additional file 3: Table S2). Since the data were derived from the same platform (i.e. Affymetrix) and processed through the same pipeline, these terms should be suitable for meta-network construction.

Taking the number of validated miRNAs and the volume of Affymetrix microarray experiments into consideration, we eventually selected 12 species to be included in the first version of our database. These species are *Arabidopsis thaliana*, *Glycine max*, *Zea mays*, *Oryza sativa*, *Gossypium hirsutum*, *Citrus sinensis*, *Medicago truncatula, Populus trichocarpa*, *Solanum lycopersicum*, *Saccharum officinarum*, *Vitis vinifera* and *Triticum aestivum*. Table 1 lists the miRNAs, targets and experiments hosted in PMTED with a detailed experiment list in Additional file 1: Table S1.

### Consensus method

#### Differentially expressed genes and meta-data connection

Genes that were differentially expressed among treatments were determined using the Linear modeling (limma; [13]), a build-in function of the program Robin, with the threshold of log-fold change as 1 and *p* value as 0.05. For meta-analysis, experiments of similar biological processes were linked with each other by common terms. The strength of the link was marked by the largest number of coefficient of variation (CV) calculated from the expression values in or among associated experiment(s). CV was used to eliminate the deviation of the units and mean values of corresponding experiments. The formula is as following:

$$\mathrm{CV}=\sigma /\left|u\right|,$$

and

$$\sigma =\sqrt{\sum {\left(\mathrm{xi}-u\right)}^{2}/\left(n-1\right)},u=\left(\sum \mathrm{xi}\right)/n$$

Where *n* is the number of samples, and *xi* is the value of *i*th sample.

### Function modules

#### Basic information

This section is for querying information about predicted target genes, such as sequence, miRNA alignment and location, gene ontology, and expression profiles in the related experiments. For easy access, four entries were provided to the users: (1) *By miRNA:* Browse or search a known miRNA and then its targets and associated experiments; (2) *By experiment*: Select an experiment of interest and retrieve expression data of target genes of a miRNA of interest; (3) *By target gene ID*: Browse or search of a target gene of a user’s interest and hence the experiments containing its expression patterns. (4) *By target prediction*: This entry is for a novel miRNA defined by a user. Targets were firstly predicted and then the expression patterns of these genes in the associated experiments can be retrieved. The default quality score of prediction is set as 4 which we consider as a stringent threshold; but users have a wide range to choose (0–8) and can select more relaxed conditions such that more candidate targets can be returned.

#### Meta-network

This module enables users to search and browse the result of a meta-analysis. The search function allows combinatorial queries of condition terms, species, and miRNA family names. Results were displayed in table format with cross references for additional species and experimental conditions containing the same miRNA(s). The browse function provides direct access to meta-data via interactive graphs generated by the Cytoscape web program [14]. There are three entries presenting different levels and angles of the curated meta-networks: (1) *By miRNA* presents users with all the experimental conditions of a selected miRNA family and associated experiments and their details; (2) *By condition* shows all the miRNAs with experiments containing their target genes that are associated with the selected condition; (3) *By bio-process* presents all conditions associated with user selected bioprocess and then the miRNAs with experiments containing their target genes. Meta-data structures provide global views of differentially expressed genes centering a miRNA family, a condition, a bioprocess or a species, where hypothesis can be created for experimental validation.

## Utility

### Basic information query by miRNA

**Figure 2:**
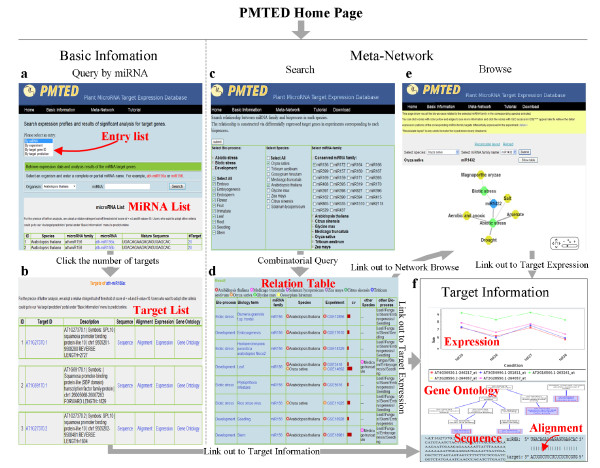
**PMTED Utilities. a**, Web page for basic information query by miRNA. **b**, List of ath-miR156a targets. **c**, Web page of meta-network search. **d**, Result table of a meta-network search. **e**, Web page for meta-network browse. **f**, Category plot of target expression and hierarchical tree of Gene Ontology.

We show the functions in Basic Information using “By miRNA” as an example. The query function confers fuzzy query capability which accepts both complete and partial miRNA names as input. It initially gives a full list of miRNAs in the species selected. As shown in Figure 2a, clicking the hyperlink on the number of targets will lead users to a detailed target list. There are seven columns in the result table. The first column contains incremental numbers. The second column is the target gene ID. The hyperlink on Arabidopsis gene ID will lead users to The Arabidopsis Information Resource (TAIR; http://www.arabidopsis.org/), a well-annotated database dedicated to Arabidopsis with much more detailed information. The third column shows gene annotation. The forth one contains target sequences in which the miRNA target loci are shown in green. In the fifth column, hyperlinks lead users to the alignment of target and miRNA. Hyperlinks on “Expression” in the sixth column give users the expression of the target and the last column shows users the target in the context of Gene Ontology hierarchy (Figure 2f).

### Meta-network search

This module enables users to perform a flexible combinatorial query of annotated bioprocesses, species, and miRNA family names for meta-analysis (Figure 2c). Results are displayed in table format which contains the list of selected objects, such as bioprocess, biology term, miRNA family name, species, experiments and the strength of relation (by CV values; Figure 2d). The presence of multiple terms demonstrates meta-relationship among them and can be considered as putative biological properties for selected miRNAs. This information can assist in raising novel hypothesis for experimental validation. For example, when we search miR156 for Biotic stress and Development in rice (*Oryza sativa*) and Arabidopsis, we found that miR156 targets are associated with leaf development in both rice and Arabidopsis. They are also related to stem, seedling and embryogenesis development in Arabidopsis. In addition, our results suggest that miR156 targets are associated with biotic stress responses, such as rice stripe virus and powdery mildew in Arabidopsis and rice. Since miRNA156 has been reported to be involved in virus-induced biological stress response in tobacco and Arabidopsis [1], results from this database search may provide a valuable clue for probable roles of miR156 as well as its targets in rice disease resistance.

### Meta-network browse

Users can browse the meta-network via three entries: by a miRNA name, by an experimental condition or by a biological process. Unlike the *search* function, meta-network can be browsed one each time to make the results easier to follow. The result is displayed by the program Web Cytoscape. As shown in Figure 2e, nodes of different types or levels of biological terms are represented with different colors. The graphical network can be manipulated in a number of features:

(1)  Click on the edges to check CV value or fold changes of targets.

(2)  Draw a node to place it in a more prominent position. We also provide a “Recalculate layout” button to make the results more clearly structured, especially after users append additional child nodes.

(3)  “Reload” button enables users to reload a picture.

(4)  Click on leaf nodes (those without out edge) can add more child nodes.

(5)  Click on an experiment node will give detailed expression profiles of the related target genes in the category plot.

For example, when we select to browse by miRNA and choose *Oryza sativa* and miR1432, a network of osa-miR1432 and five nodes-- aerobic and anoxic, *Magnaporthe oryzae*, drought, arsenate and salt was obtained. This result is consistent with the function of predicted rice miR1432 target, a calmodulin-binding protein, and suggests a role of this miRNA and probably associated targets in calcium signaling [15]. This rice gene has been found to be differentially regulated in response to drought stress [16]. The information obtained by clicking the five bio-condition nodes will provide further related experiments and then target expression profiles which may help in further experimental validation.

## Discussions

Since the emergence of plant miRNAs as important regulators for various biological processes, a number of databases have been designed to host miRNAs [17-19], together with various target prediction programs [11,20-22]. For example, miRBase hosts both plant and animal miRNAs and their precursor sequences [2,19,23], while PMRD, a specialized plant miRNA database, integrates plant miRNA data curated from recent literatures with functions for sequence information, secondary structure, and target gene retrieval [9]. PMTED takes advantage of the extant gene expression patterns in the high volume of microarray data generated in the past two decades, providing a platform for plant researchers to infer miRNA functions through their target performance. Furthermore, the expression patterns among experiments with the same target genes were linked together by curated common biological terms allowing for a global meta-analysis of the experiments. The Affymetrix platform provides a standardized system with a high degree of reproducibility [24,25]. Despite this, the quality of the experiments is variable and needs manual screening in terms of the design and the completeness. The experiments we used were carefully screened and processed with stringent criteria set in the Robin program [7]. After rigorous filtering, the retained microarray experiments were of high quality with clear biological object and experimental design. To ensure high quality target gene prediction, our pipeline followed the stringent rules as Allen et al. (2005) which were derived from a set of experimentally validated miRNA-target pairing rules. With a score of ≤ 4, the program has a false negative rate of 0.03 [10].

PMTED boasts a number of querying and analysis functionalities developed to facilitate functional discovery, suitable for both validated miRNAs and potential novel miRNAs designated by users. In the mean time, the connection of miRNAs with differentially expressed targets in a number of microarray experiments allows a cross species/experiments/bioprocess meta-analysis. The contextual information of target gene expression data should be indicative of potential biological functions and the regulatory network that a miRNA involves, facilitating the process of biological discovery. The current version of PMTED focuses on abiotic, biotic and development experiments and should expand its scope in the near future. Despite the appearance of new technologies for gene expression study, such as RNA-seq, microarray analysis remains as an effective option for genome-wide gene expression analysis. With further accumulation of the microarray data, more species will be included. Pipelines are being developed for data compatibility from other microarray platforms. Experiments from next generation sequencing technology such as RNA-seq data, expression data of miRNAs themselves, as well as related literatures will also fall in the scope of collection for the future version of PMTED.

## Conclusions

PMTED is a database that comprises a number of querying, analytical, and visualization functionalities for miRNAs targets. It is designed to link miRNA target gene expression patterns from various microarray experiments and reveal contextual significance of miRNA-target gene regulatory networks. PMTED should be a useful resource for researchers working in both model plants and agriculturally important crops.

## Availability and requirements

Database name: PMTED

Database homepage: http://pmted.agrinome.org

Browser requirement: the application is optimized for Internet Explorer 9, Mozilla FireFox 16.0.2 and Safari 5.1.7.

Datasets in PMTED is freely available. Please use the link ‘Contact Us’ on the PMTED homepage or email Dr. Long Mao at maolong@caas.cn to request specific data subsets.

## Competing interests

The authors declare that they have no competing interests.

## Authors’ contributions

YL and LM conceived the study. XS, DL, BD, RZ, AL, WD, NS and LM analyzed the data, XS and LY developed the database. BD helped process the data. XS, YL, and LM wrote the paper. All authors read and approved the final manuscript.

## Supplementary Material

Additional file 1: Table S1 Experiment list. Click here for file

Additional file 2: Figure S1 Comparison of target prediction results. A, Numbers of targets predicted by PMTED and psRNATarget. B, Numbers of targets validated by degradome data when compared with those predicted by PMTED or psRNATarget. Click here for file

Additional file 3: Table S2 Statistic data for meta-analysis. Click here for file
