# Supplementary material for: PMTED: a plant microRNA target expression database
Source: BMC Bioinformatics. 2013 Jun 3;14:174. doi: 10.1186/1471-2105-14-174 (PMC3680227; doi:10.1186/1471-2105-14-174)
Supplement: Additional file 1: Table S1 — Experiment list. [file 1471-2105-14-174-S1.pdf]

Additional file 1:

**Table S1. Experiment list.**	
Stars(*) after geo_accession number means the corresponding experiments were selected for meta-analysis.	
**GEO_ Accession**	**Title**
Arabidopsis thaliana	
GSE10019	Identification of RGA downstream genes by using steroid-inducible system
GSE10323	Testing Arabidopsis for the presence of arbuscular mycorrhizal signalling pathways
GSE10414	microRNA156 resistant SQUAMOSA-PROMOTER-BINDING PROTEIN-LIKE13 (mSPL13)
GSE10496	Expression analysis of the effect of protoplasting and FACS sorting in roots exposed to iron deficiency (-Fe)
GSE10497	Expression analysis of root developmental zones after iron deficiency (-Fe) treatment
GSE10501	Expression analysis of root cell-types after iron deficiency (-Fe) treatment
GSE10502	Time course expression analysis of the iron deficiency (-Fe) response in Arabidopsis roots
GSE10568	camta3 mutant versus wild type microarray analysis
GSE10643	Transcription profiling of Arabidopsis dor mutant and wild-type plants in response to drought stress.
GSE10646	BTH treated mkk1, mkk2 and mkk1/2 knockout mutant
GSE10670	Global expression profiling of wild type and transgenic Arabidopsis plants in response to water stress
GSE10719	Response of Arabidopsis cell culture to phytoprostane A1
GSE10732	Identification of TGA-regulated genes in response to phytoprostane A1 and OPDA
GSE10749	Response of Arabidopsis cell culture to cyclopentenone oxylipins
GSE10801	C. fulvum Avr2
GSE10812	Expression data from thylakoidal ascorbate peroxidase overexpressor Arabidopsis thaliana (Col) rosette leaves
GSE11119	SOL2 mutation affect gene expresstion at root apex
GSE11216*	Brassinazole treatment of arf2 and wild-type dark-grown seedlings
GSE11250	Overexpression of miR396
GSE11505	The Arabidopsis BRAHMA Chromatin Remodelling ATPase Is Involved in Direct Repression of Embryonic Traits in Leaves
GSE11532	Octopus transcriptome (affy_phloem_ath1)
GSE11538	The association between gene expression and whole genome histone H3K4 mono-, di, and trimethylation: expression data
GSE11758	Plant response to misfolded protein in the cytosol
GSE11807	Flg22 regulates MAP kinase 6 interaction with an ethylene response factor substrate via ethylene
GSE11852	Effect of uniconazole on wt and pkl mutant germinating seeds
GSE12029*	NFYA5, a CCAAT binding transcription factor important for drought resistance in Arabidopsis
GSE12401	Transcript abundance data from seedlings of wild-type Ws and ged1 (greening after extended darkness 1) mutant
GSE12402	Expression data from Arabidopsis seed compartments at the pre-globular stage
GSE12522	Comparison of gene expression between wild type and gl3 egl3 trichomeless mutant green seedling tissues
GSE12551	Inducible expression of trichome regulators, GL1 and GL3
GSE12619*	Heat shock response of til1-1 mutant plants
GSE12676	Arabidopsis thaliana Ler developmental series
GSE12715	ABA and ethylene signaling crosstalk
GSE12856*	Penetration resistance: Wildtype and ataf1-1 mutant response to Bgh 12 h after inoculation, 2*2 factorial design

GSE12964*	Auxin stimulates brassinosteroid biosynthesis in Arabidopsis roots
GSE13596	A gene expression map of Arabidopsis thaliana shoot apical meristem stem cell niche
GSE13803	Interaction between the light environment and the Arabidopsis wound response
GSE13833*	Transcriptome changes triggered by the synthetic defense elicitors DCA and INA in Arabidopsis thaliana
GSE13881	Transcriptional profiles between mp mutant seedlings and transgenics carrying the dexamethasone-inducible GR-bdl protein
GSE13913	WT and php mutants
GSE13929	Arabidopsis thaliana three hours after infection with Agrobacterium tumefaciens
GSE13930*	Arabidopsis thaliana six days after infection with Agrobacterium tumefaciens
GSE14106	Transcriptome data from inflorescence stalks of intact A. thaliana plants after infection with A. tumefaciens
GSE14229	The differentially expressed genes identified in the microarray analysis using myb5 and wild-type (Col) seeds
GSE14247	Interplay between ethylene, ETP1/ETP2 F-box proteins, and degradation of EIN2 triggers ethylene responses in Arabidopsis
GSE14374*	Expression data from Col-0 and pil5 imbibed seeds after far-red or far-red/red light irradiation.
GSE14420	Global gene expression of Arabidopsis lines with altered ANAC102 expression under normal and low oxygen conditions
GSE14493	Immunopurified mRNA-ribosome complexes expose cell-type specific plasticity during hypoxia in Arabidopsis root tips
GSE1491*	Identification of Inhibitors of Auxin Transcriptional Activation via Chemical Genetics in Arabidopsis
GSE14961	Expression data from Arabidopsis Seedlings treated with Salicylic Acid
GSE15160	Expression data from Arabidopsis seed compartments at the heart stage.
GSE15165	Expression data from Arabidopsis seed compartments at the mature green stage.
GSE15189	Early Iron Deficiency Induced Changes in Arabidopsis Roots
GSE15577	Involvement of the Chromatin Modifier ATX1 and of the Arabidopsis Myotubularin Homolog (MYO1) in the Response to Drought
GSE15649	Early phosphate-deficiency induced signal changes in Arabidopsis roots
GSE15680	Laser microdissection of Arabidopsis cells at the powdery mildew infection site
GSE15689*	A complementary role for ELF3 and TFL1 in the regulation of flowering time by ambient temperature.
GSE16061	Transcriptome comparison of Col-0, se-1, ago1-27 and se-1 35S::MIR156 in Arabidopsis thaliana.
GSE16222	Effects of heat, anoxia, and combined heat-anoxia treatments
GSE16472	Identification of early flg22 responsive genes in Arabidopsis mesophyll cells
GSE16497	Arabidopsis thaliana gene expression changes upon treatment with green peach aphid saliva
GSE16557	Gene expression changes underlying initial cellular and molecular responses to MAMPs in Arabidopsis mesophyll cells
GSE16722	Expression data from Arabidopsis thaliana under phosphate starvation stress
GSE16765*	Transcriptomic and phenotypic variation for salt stress response in Arabidopsis
GSE16964*	Iron-deficiency-induced changes in wild type, ubc13A and cucumber CsUBC13 overexpressed Arabidopsis roots
GSE17193*	Transcript profile of chitosan-treated Arabidopsis seedlings
GSE17343	Expression data from Arabidopsis pollen and semi in vivo- and in vitro-grown pollen tubes.
GSE17382*	Identification of early flg22 responsive genes in Arabidopsis seedlings
GSE17499*	Expression data from 2-week-old Arabidopsis untreated seedlings grown under a short day condition
GSE17610*	Gene expression in response to AGL15 during somatic embryogenesis
GSE17824	Transcriptional profiling after inhibition of cellulose synthesis by TA and IXB in Arabidopsis thaliana suspension cells

GSE17845*	Transcriptional profiling of leaf blades and petioles subjected to shade avoidance syndrome
GSE18071*	Chloroplast polynucleotide phosphorylase null mutant (pnp1-1) and phosphate starvation
GSE18153*	Effect of deuterium oxide on Arabidopsis gene expression
GSE18217	Global gene expression analysis of transgenic, mannitol-producing, and salt-tolerant Arabidopsis thaliana indicates widespread changes in abiotic and biotic stress-related genes
GSE18311*	Comparison of gene expression in wild type and angustifoila3 leaf primorida
GSE18329*	Transcriptome changes triggered by Hyaloperonospora parasitica arabidopsis Noco2 in WT and wrky72 mutants at 96 hpt
GSE18417	Arabidopsis transcriptome changes caused by ectopic production of a turtle riboflavin-binding protein
GSE18624	H2A.Z - Containing Nucleosomes Mediate the Thermosensory Response in Arabidopsis
GSE18666	Persistent heat stress in Arabidopsis
GSE18928*	Expression data from 5-week-old Arabidopsis
GSE18960	Gene expression changes in Arabidopsis thaliana treated with Myzus persicae saliva
GSE18980	Transcriptome of Arabidopsis APS kinase null mutants
GSE18981*	Analysis of RLI on RNA stability in A. thaliana
GSE18982	Molecular responses to external pH changes in roots of Arabidopsis thaliana
GSE18984	Treatment of Arabidopsis with low concentration of nitrate
GSE18986	Transcriptome of the exo70A1 mutant
GSE18987	Transcriptome analysis during axillary shoot outgrowth
GSE19241	A novel S-sulfocysteine synthase essential for chloroplast function in Arabidopsis thaliana
GSE19242*	Mitochondrial beta-cyanoalanine synthase is essential for root hair formation in Arabidopsis thaliana
GSE19273	Biological function of Pip3
GSE19700	Time of day shapes the Arabidopsis drought transcriptomes
GSE19978	Different gene expressions in root tips between CLE10 and CLE25 treatment
GSE20044	High resolution NO3 response of Arabidopsis Roots
GSE20188	Expression data in response to neonicotinoid insecticides
GSE20221	Cellulose synthase mutants
GSE20222	Cesium Toxicity in Arabidopsis
GSE20223	Gibberellin (GA) Treatment of Arabidopsis Root Tips
GSE20226*	Arabidopsis thaliana/Phytophthora parasitica compatible interaction transcriptome
GSE2133	Effects of anoxia and sucrose on seedling growth
GSE2406	WTv.AOXantisense
GSE3350	SLR/IAA14-dependent auxin induced lateral root initiation
GSE3416*	Diurnal gene expression in Arabidopsis thaliana Col-0 rosette leaves
GSE3423*	Carbon fixation (endogenous sugar) and light-dependent gene expression
GSE4847	Expression data from tocopherol deficient seedlings of Arabidopsis
GSE5526	Transcriptional Programs of Early Reproductive Stages in Arabidopsis
GSE5529	Arabidopsis E2F target genes
GSE5530*	Hydrogen peroxide stress and Zat12 over-expression in Arabidopsis.
GSE5534	Response to cold, plate grown plants
GSE5535	Response to cold, soil grown plants
GSE5539	Over-expression of MBF1c enhances stress tolerance
GSE5611*	Differential gene expression patterns in the phosphate deficient mutant, pho 1
GSE5614	Changes in Gene Expression in Brassica oleracea Shoots during Phosphate Starvation (Cross-species study)
GSE5616*	AtGenExpress: Response to Phytophthora infestans

GSE5621	AtGenExpress: Stress Treatments (Cold stress)
GSE5622	AtGenExpress: Stress Treatments (Osmotic stress)
GSE5623	AtGenExpress: Stress Treatments (Salt stress)
GSE5625	AtGenExpress: Stress Treatments (Genotoxic stress)
GSE5629	AtGenExpress: Developmental series (seedlings and whole plants)
GSE5631	AtGenExpress: Developmental series (roots)
GSE5634	AtGenExpress: Developmental series (siliques and seeds)
GSE5687	AtGenExpress: Different temperature treatment of seeds
GSE5688	AtGenExpress: Response to sulfate limitation
GSE5696	AtGenExpress: Effect of brassinosteroids in seedlings
GSE5697	AtGenExpress: Comparison of plant hormone-related mutants
GSE5701	AtGenExpress: Basic hormone treatment of seeds
GSE5712*	Transcriptome analysis of ARRESTED DEVELOPMENT 3 mutant.
GSE5722*	Functional Genomics of Ozone Stress in Arabidopsis.
GSE5729	Role of COV in vascular patterning
GSE5736	To identify changes in gene expression during silique senescence in Arabidopsis thaliana
GSE5740	Polycomb Binding Protein
GSE5745	Mutant array
GSE5751	The early post-germinative embryo and endosperm transcriptomes in Arabidopsis
GSE5759	red illumination w/o lincomycin
GSE6151	The mechanisms involved in the interplay between dormancy and secondary growth in Arabidopsis
GSE6160	Differential gene expression patterns in potassium-starved and caesium-treated plants
GSE6165*	The effect of mutations in AtrbohC on the pattern of gene expression in primary root tissue.
GSE6179	An investigation into transcriptional changes in developing Arabidopsis leaf caused by novel signalling protein, SPH1.
GSE6181	Assembly of the cell wall pectic matrix.
GSE629*	Auxin-mediated gene expression in WT, iaa17, axr3 and iaa5iaa6iaa19 mutants
GSE631	Auxin mediated gene expression in WT and arf2-6 mutant
GSE6516	Silverleaf whitefly 2nd instar feeding on 7-week old Arabidopsis thaliana rosette leaves
GSE6696	Transcriptome analyses show changes in gene expression to accompany pollen germination and tube growth in Arabidopsis
GSE6831	Systemic response to avirulent bacterial infection
GSE6832	Cytokinin treatment on aerial parts of seedlings
GSE6954	Identification of AGL24 downstream genes by using XVE inducible system
GSE7112	Abscisic acid effect on wild type and the abh1 mutant
GSE7227	microRNA160 resistant AUXIN RESPONSE FACTOR10 (mARF10) germinating seeds
GSE7334	Microarray Analysis of Arabidopsis Genome Response to Aluminum Stress
GSE7353*	Early GA response genes in Arabidopsis thaliana
GSE7432*	Ethylene and auxin interactions in the roots of Arabidopsis seedlings
GSE7636	Expression analysis of the effect of protoplasting and FACS sorting in roots
GSE7639	Expression analysis of root developmental zones after treatment with salt
GSE7641	Expression analysis of root cell-types after treatment with salt
GSE7642*	Time course expression analysis of the salt stress response in Arabidopsis roots
GSE8279	Transgenerational Stability of the Arabidopsis Epigenome Is Coordinated by CG Methylation
GSE8739	Early gibberellin responses in Arabidopsis

GSE8912*	Global expression profiling to study the effect of sulfometuron methyl herbicide treatment on Arabidopsis thaliana
GSE8913*	Global expression profiling to study the effect of primisulfuron herbicide treatment on Arabidopsis thaliana
GSE8925*	Global expression profiling to study the effect of imidazolinone herbicide treatment on Arabidopsis thaliana
GSE8926*	Global expression profiling to study the effect of triazolopyrimidine herbicide treatment on Arabidopsis thaliana
GSE8927	Global expression profiling to study the effect of glyphosate herbicide treatment on Arabidopsis thaliana
GSE8951*	A light-independent allele of phytochrome B faithfully recapitulates photomorphogenic transcriptional networks
GSE9148*	Expression data of 10-day-old wild-type and chl1-5 plants exposed to 25 mM nitrate for 0h or 0.5h
GSE9311	Gene expression in roots and shoots of plants grown on selenate
GSE9605	Target genes of AGAMOUS during early flower development in Arabidopsis
GSE9728	COP9 signalosome (csn) mutant analysis
**Citrus sinensis**	
GSE10798*	Transcriptional analysis of the sweet orange interaction with the citrus canker pathogens
GSE29633	Expression data of sweet orange in response to Candidatus Liberibacter asiaticus infection at advanced stage
GSE30502*	Transcriptional response of susceptible and tolerant citrus to infection with Candidatus Liberibacter asiaticus
**Glycine max**	
GSE10251	Gene expression profiling implicates novel hormonal regulation of the floral initiation process in soybeans
GSE10284	Detection and Validation of Single Feature Polymorphisms in Cowpea using a Soybean Genome Array
GSE10730	Analysis of Iron Deficiency in Soybean Leaf Tissue
GSE12286	Genomic Expression Profiling of Mature Soybean (Glycine max) Pollen
GSE12300	Suppression of oleosin in soybean cotyledon
GSE12314	Storage Protein Suppression in Transgenic Soybean Cotyledons
GSE17883	Global transcriptome profiling of wild soybean (Glycine soja) roots under NaHCO3 treatment
GSE18822	Transcript profiling of common bean using the Soybean Genome Array: optimizing analysis by masking biased probes
GSE20323*	Alkaline-stress response in Glycine soja leaf identifies specific transcription factors and ABA-mediated signaling factors
GSE20972	Alterations in soybean gene expression profile after foliar application of lipo-chitooligosaccharide (LCO) from Bradyrhizobium japonicum under sub-optimal temperature
GSE22158	Expression of the Stress-Related Genes for Glutathione S-Transferase and Ascorbate Peroxidase in the Most-Glycinin-Deficient Soybean Cultivar Tousan205 during Seed Maturation.
GSE22978	Microarray Analysis of Phytophthora sojae Gene Expression During Early Infection
GSE26198*	Comparative physiology and transcriptional networks underlying the heat shock response in Populus trichocarpa, Arabidopsis thaliana and Glycine max [Soy]
GSE26208	Expression data from the seed coats of black (iRT) and brown (irT) soybean variant for alleles of the R locus
GSE26443	Characterization of gene expression profile in developing soybean seeds by DNA microarray
GSE27894	Expression data of Soybean root apical meristem and leaf
GSE29653	GmMPK4 is a negative regulator of SA-mediated defense response
GSE7108	Gene expression analysis in soybean with respect to Pahakopsora pachyrhizii at V2 growth stage
GSE7511	Expression data from soybean seed compartments with embryos at the heart stage
GSE7592	Expression data from SRB embryo regions at the globular stage

GSE7881	Expression data from soybean seed compartments with embryos at the cotyledon stage
GSE9374	Exploration of microarrays as tools to assess substantial equivalence of genetically modified soybeans
GSE9730*	Effect of foliar spray of lipo-chitooligosaccharide (Nod factor) on soybean leaf gene expression.
**Gossypium hirsutum**	
GSE16467	Global gene expression analysis of waterlogging stressed Cotton root and leaf tissue
GSE26522*	Transcription profiling of One month old Gossypium herbaceum plant leaf during drought and watered condition
**Medicago truncatula**	
GSE13602*	Transcriptome Profiles of Medicago Stem Internodes
GSE13921	Medicago truncatula germinated seedlings grown in Petri dishes for 3 days then carry out salt stress
GSE17253*	affy-agro-bi-medicago-AGRO-BI-Toulouse
GSE18318	affy_root-dvt-nitrogen_medicago: Genetic determinism of root development in Medicago truncatula
GSE20587*	Comparison of plant responses upon Aphanomyces euteiches infection in wild type (A17) and NFP allelic mutants
GSE22835	Characterization of a Medicago truncatula P450 monooxygenase mutant
GSE23721	Expression data from Sinorhizobium meliloti strains Rm8530 and Rm8530 mucR.
GSE25034	Molecular analysis of heterosis in alfalfa
GSE8115	A transcript profile of Medicago truncatula root meristem cells
GSE8131*	A transcript profile of Medicago truncatula 2HA and Jemmalong during somatic embryogenesis
GSE8138	A transcript profile of Medicago truncatula 2HA tissue culture with auxin treatment
**Oryza sativa**	
GSE10054	Expression information of splicing factor OsSKIPa knock-down and overexpressed rice
GSE10857	Gene expression of rice root tips before, at and buckled by a hard layer in two rice varieties
GSE11025*	Comparative transcriptional profiling of two contrasting rice genotypes in response to rice stripe virus infection
GSE11175*	Comparison of transcriptome profile between wild-type and dst mutant plants
GSE11966	Expression data from rice embryo,endosperm,root,leaf and seedling
GSE12069	Mycroarray analyses reveal that plant mutagenesis may induce more transcriptomic changes than transgene insertion
GSE13735*	Expression data from rice genotypes FL478 and IR29
GSE14275*	Expression data for heat shock in rice seedlings
GSE14298	Rice expression atlas (2): Pollination - Fertilization
GSE14300	Rice expression atlas (4): Vegetative tissues
GSE14403*	Root-specific transcriptional profiling of contrasting rice genotypes in response to salinity stress
GSE14692*	Temporal regulatory role of miR156 during leaf development
GSE15046	Transcriptome analysis of gibberellin-signaling mutants in rice [Oryza sativa]
GSE15448*	Glycinebetaine-induced water-stress tolerance in codA-expressing transgenic indica rice
GSE15912	Whole genome transcriptome profiling of Pusa 1266 and Pusa Basmati 1 in panicle primordia stage
GSE16108	Transcription profiling of parental lines and bulked salt sensitive and salt tolerant RILs derived from 2 rice varieties
GSE16265	SNEP: Simultaneous detection of nucleotide and expression polymorphisms using Affymetrix GeneChip
GSE17169	bulked RILs with high and low grain number per panicle derived from 2 cultivars at panicle primordia stage
GSE17194	Genome-wide gene expression profiling of rice Indica cultivar Zhongxian 3037 and mutant phoenix (pho) panicle
GSE17245	Transcriptome analysis of iron and phosphorus interaction in rice seedlings

GSE18361*	Temporal gene expression analyisis from rice root (cv. Nipponbare) infected with Magnaporthe oryzae strain Guy11
GSE18930*	Microarray data from aerial tissue of submergence tolerant M202(Sub1) and intolerant M202
GSE19239	Transgenic rice line carrying the maize resistance gene Rxo1 to Xanthomonas oryzae pv. oryzicola
GSE19844*	affy_xoo_rice-Transcriptomics-based identification of Xoo strain BAI3 Talc targets in rice
GSE20522	Genome-wide gene expression of rice cultivar Gui-630 (Indica) X Taiwanjing (Japonica) and mutant lemmata (lem) panicle
GSE24048*	Expression data from field droughted rice plants
GSE24228	Identification of Rhizome-Specific Genes by Genome-wide Differential Expression Analysis in Oryza longistaminata
GSE25073	Expression data from rice seedlings of wild type and the CHR729 mutant
GSE27726	Analysis of anther transcriptomes to identify genes contributing to meiosis and male gametophyte development in rice
GSE27988	genome-wide gene expression profiling of rice pollen in defferent developmetn stages
GSE30818	Transcript profiling of crown rootless1 mutant stem base reveals new elements associated with crown root development in rice
GSE31834	Expression data from shoot bases of rice seedling after gravistimulation
GSE4471*	Expression data from rice varieties Azucena and Bala grown in 0 and 1ppm arsenate
GSE5167*	Rice seedling hormone treatment
GSE6719	Cytokinin responsive genes in rice
GSE6720	Gene expression in OsRR6-overexpression line
GSE6901*	Expression data for stress treatment in rice seedlings
GSE6908*	Transcript Profiling of the Aerobic and Anoxic Rice Coleoptile
GSE7197	Down-regulation of OsSRT1 induces DNA 閿槈浮ragmentation and cell death in rice
GSE7256	Identification of rice genes differentially expressed upon virulent infection by Magnaporthe grisea
GSE8216	Transcriptional profiling of Cellulase induced hypersensitive reaction like response in rice
GSE8380	Rice Gene Network Inferred from Expression Profiling of Plants Overexpressing OsWRKY13
GSE9498	Global gene expression profiles of Oryza sativa wild type Zhonghua11 and mutant gif1 in filling stage
**Populus trichocarpa**	
GSE17804	Habituation to thaxtomin A provides enhanced and durable resistance to inhibitors of cellulose synthesis
GSE21480	Novel aspects of transcriptional regulation in the winter survival and maintenance mechanism of perennial woody plants, poplar.
**Saccharum officinarum**	
GSE11934	Differential expression of genes in the leaves of sugarcane in response to sugar accumulation
GSE5021	Transcripts associated with cell wall metabolism and development in the sugarcane stem by GeneChip expression profiling
**Solanum lycopersicum**	
GSE14358*	Gene regulation in parthenocarpic tomato fruit
GSE14637	Expression data of tomato fruit responses to Botrytis cinerea
GSE16401*	tomato salt response
GSE19787	Expression data from PSARK::IPT Nicotiana and wildtype plants
GSE19792	Transcript profiling of tomato mutants producing anthocyanins in the fruit
GSE21020	Tomato root transcriptome response to a nitrogen-enriched soil patch
GSE21999*	Tomato fruit wound inoculation by C. coccodes and ammonium treated
GSE22304	Expression data in response to abiotic stresses in tomato at flowering stage
GSE23626	Genome wide expression analysis of psaA and psbA tobacco mutant plants.

**Triticum aestivum**	
GSE12936*	Transcriptomic analysis of the effect of silicon on wheat plants infected or uninfected with powdery mildew
GSE13660*	Gene expression analysis of the wheat response to infection by Fusarium pseudograminearum
GSE15864	Metabolism-based resistance to herbicides in black-grass
GSE16457	affy_seed_kinetic_wheat-Transcriptomic wheat seed
GSE21386	RNA profiling of Fusarium head blight-resistant wheat addition lines containing the Thinopyrum elongatum chromosome 7E
GSE22080*	Pleiotropic expression of endogenous genes upon fungus infection of wheat plants containing anti-fungal transgenes
GSE27320	Expression data in wheat (T. aestivum L.) near isogenic lines in response to powdery mildew infection
GSE31760*	Transcription profiling wheat responses to adapted and non-adapted isolates of the blast fungus, Magnaporthe
GSE31761	Transcription profiling of wheat interacting with incompatible and compatible yellow rust in a Yr1-containing genotype
GSE31762	Wild emmer wheat comparison of drought resistant vs. susceptible genotypes under terminal drought
GSE4929	wheat expression level polymorphism study parental genotypes 2 biological reps
GSE5937	Wheat expression level polymorphism study parental genotypes 2 biological reps from SB location
GSE6027	Microarray expression analysis of meiosis and microsporogenesis in hexaploid bread wheat
GSE9767	Genotypic differences in water soluble carbohydrate metabolism in stem
**Vitis vinifera**	
GSE11857	Gene expression patterns associated with grapevine resistance to downy mildew mediated by the Rpv1 and Rpv2 genes
GSE27180	Expression data from micropropagated Vitis vinifera when transferred to ex vitro conditions
GSE31662	Grape skin transcriptome in the berries cultured in vitro treated with exogenous abscisic acid
GSE31664	Grape skin transcriptome in the berries grown on the vine treated with exogenous abscisic acid
**Zea mays**	
GSE10023*	Maize gene expression during infection with Ustilago maydis
GSE10236*	Similar patterns of additive and non-additive gene expression in maize hybrids with varying levels of heterosis
GSE10237	Gene expression variation among eight maize inbreds
GSE10243	Profiling expression changes caused by a segmental aneuploid in maize
GSE11531	Downregulation of cinnamoyl-coenzyme A reductase in maize (affy_ccr_maize)
GSE12892	Maize gene expression during infection with Ustilago maydis strain SG200Dpep1
GSE15048*	Gibberellin-induced gene expression in maize mesocotyl under deep-sowing condition
GSE15371	Auxin-induced gene expression in mesocotyl elongation of maize inbred line 3681-4 tolerant to deep-sowing
GSE16567*	Genome-wide transcriptome analysis of two maize inbred lines under drought stress during the seedling stage
GSE18491*	Expression data from maize endosperm
GSE18846	affy_tj_maize-Understanding the Diversity of Maize Cell Wall
GSE19212	Profiling expression changes caused by a segmental aneuploid in maize meristem tissues
GSE19501*	Maize gene expression during infection with Fusarium moniliforme
GSE19559*	Maize gene expression during infection with Ustilago maydis strain SG200鍪唂ox1
GSE21070*	Expression profile of contrasting maize genotypes grown on acid and control soil (root tips)
GSE22479*	Expression profile of contrasting maize genotypes grown in acid and control soil (leaves)
GSE22948	affy_diversity_maize-Understanding the Diversity of Maize Cell Wall
GSE22950	affy_ams_maize-Cell wall maize mutant during arbuscular mycorrhizal symbiosis

GSE24624	Understanding the Diversity of Maize Cell Wall (affy_cellwall_maize)
GSE27626	Responses of Zea mays root tissue to inoculation with the necrotrophic root pathogen Phytophthora cinnamomi
GSE28479	Maize gene expression during infection with Ustilago maydis strains SG200Dpit1 and SG200Dpit2
GSE29747*	Sporisorium reilianum Infection Changes Inflorescence and Branching Architectures of Maize
GSE31188	Maize gene expression during infection with Colletotrichum graminicola
GSE7030	Phenotypic and molecular characterisation of a novel Bt2 allele in maize
GSE8174	Cis-transcriptional variation in maize inbred lines B73 and Mo17 leads to additive expression - Seedling data
GSE8176*	Cis-transcriptional variation in maize inbred lines B73 and Mo17 leads to additive expression - Immature ear data
GSE8179*	Cis-transcriptional variation in maize inbred lines B73 and Mo17 leads to additive expression - Embryo data
GSE8188	Expression profiling of zmet2-m1 mutants relative to wild-type
GSE8194*	Cis-transcriptional variation in maize inbred lines B73 and Mo17 leads to additive expression
GSE8275*	Non-additive and imprinted gene expression in hybrid maize endosperm_13DAP
GSE8278*	Non-additive and imprinted gene expression in hybrid maize endosperm_19DAP
