# Supplementary material for: PMTED: a plant microRNA target expression database
Source: BMC Bioinformatics. 2013 Jun 3;14:174. doi: 10.1186/1471-2105-14-174 (PMC3680227; doi:10.1186/1471-2105-14-174)
Supplement: Additional file 2: Figure S1 — Comparison of target prediction results. A, Numbers of targets predicted by PMTED and psRNATarget. B, Numbers of targets validated by degradome data when compared with those predicted by PMTED or psRNATarget. [file 1471-2105-14-174-S2.pdf]

Additional file 2:

A

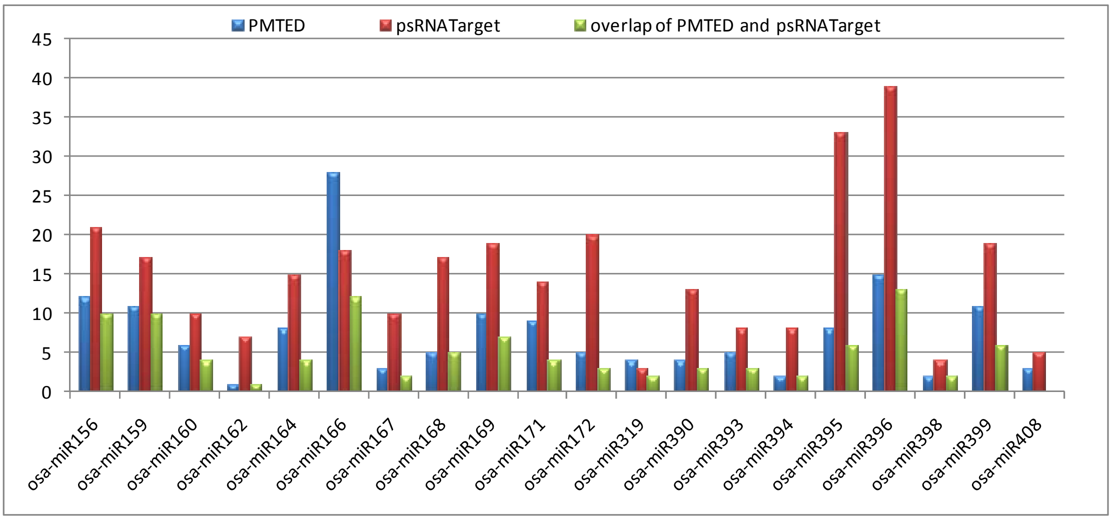

B

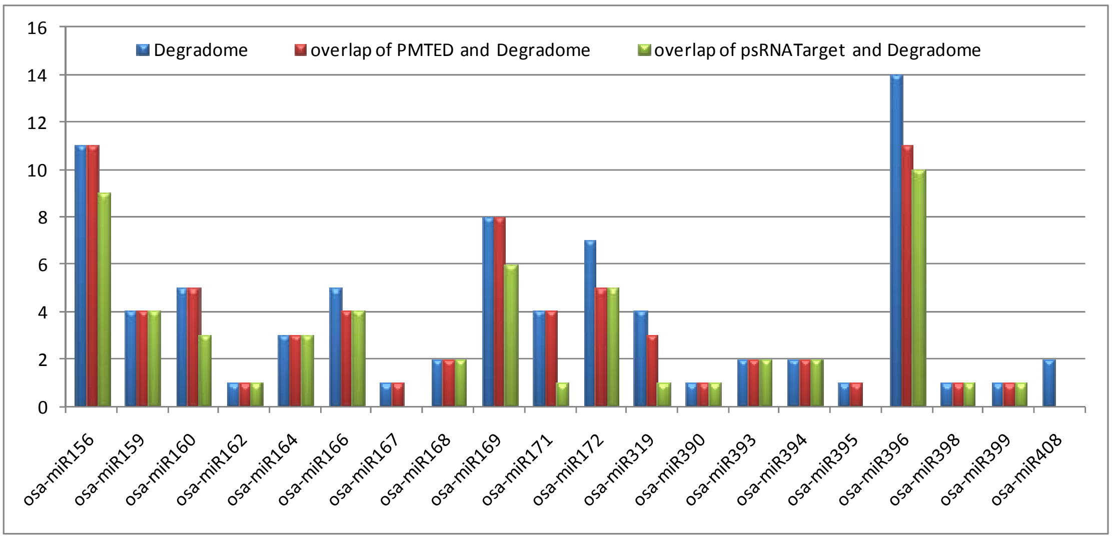

Figure S1: Comparison of target prediction results. A, Numbers of targets predicted by PMTED and psRNATarget. B, Numbers of targets validated by degradome data when compared with those predicted by PMTED or psRNATarget.
