# Supplementary material for: PMTED: a plant microRNA target expression database
Source: BMC Bioinformatics. 2013 Jun 3;14:174. doi: 10.1186/1471-2105-14-174 (PMC3680227; doi:10.1186/1471-2105-14-174)
Supplement: Additional file 3: Table S2 — Statistic data for meta-analysis. [file 1471-2105-14-174-S3.pdf]

Additional file 3:

Table S2. Statistic data for meta-analysis

Numbers in each cell means the number of differentailly expressed targets and corresponding miRNAs in the form of #miRNA/#target.

**Bio-process**	**Term**	**Species(#miRNA family/#Target)**								
		**ath**	**csi**	**gma**	**ghr**	**mtr**	**osa**	**sly**	**tae**	**zma**
Abiotic stress	Acid									12/27
	Aerobic and anoxic						59/180			
	Alkaline			36/71						
	Ammonium							3/2		
	Arsenate						51/112			
	Carbon fixation and light	28/47								
	Chitosan	14/26								
	DCA and INA	4/4								
	Deuterium oxide	18/30								
	Drought	1/1			2/1		63/204			16/32
	H2O2	14/15								
	Herbicide	39/61								
	Hormone	23/39					6/8			1/1
	Hypoxia						19/20			
	Illumination	36/70								
	Iron	8/9								
	Lipo-chitooligosaccharide			1/1						
	Nitrate	3/5								
	Nitrogen					14/21				
	Ozone	22/34								
	Phosphate	7/11								
	Salt	32/61					58/180	5/5		
	Si								13/12	
	Temperature	36/80		29/63			57/206			
	Water						9/8			
Biotic stress	Aphanomyces euteiches					29/52				
	Blumeria graminis f.sp. hordei	35/73								
	C58 and GV3101	9/13								
	Candidatus Liberibacter asiaticus		3/12							
	Citrus canker pathogens		7/19							
	Colletotrichum coccodes							7/6		
	Colletotrichum graminicola									1/1
	Flg22	8/10								
	Fungus								9/12	
	Fusarium moniliforme									8/7
	Fusarium pseudograminearum								3/2	
	Hyaloperonospora parasitica arabidopsis Noco2	17/26								
	Magnaporthe								8/12	

	Magnaporthe oryzae						43/99			
	Phytophthora cinnamomi									1/1
	Phytophthora infestans	31/48								
	Phytophthora parasitica	10/18								
	Powdery mildew								12/12	
	Rice stripe virus						13/20			
	Sporisorium reilianum									3/2
	Ustilago maydis									16/50
	Xanthomonas oryzae pv. oryzae						8/15			
Development	Embryo									7/9
	Embryogenesis	24/39								
	Endosperm									9/28
	Flower	9/17								
	Fruit							3/3		
	Immature ear									4/8
	Leaf	25/41				29/50	5/6			
	Root	13/19								
	Seedling	9/10								8/14
	Stem	7/9				24/38				
